# Supplementary material for: Mutations in PCYT2 disrupt etherlipid biosynthesis and cause a complex hereditary spastic paraplegia
Source: Brain. 2019 Oct 22;142(11):3382–97. doi: 10.1093/brain/awz291 (PMC6821184; doi:10.1093/brain/awz291)
Supplement: awz291_Supplementary_Data [file awz291_supplementary_data.zip › awz291-Suppl_data/Supplementary_Data4.zip!/Supplementary information 4 - plasma lipidomics.zip!/Plasma lipidomics/Control vs Patient2/Statistical plots and data/Volcano plot.pdf]

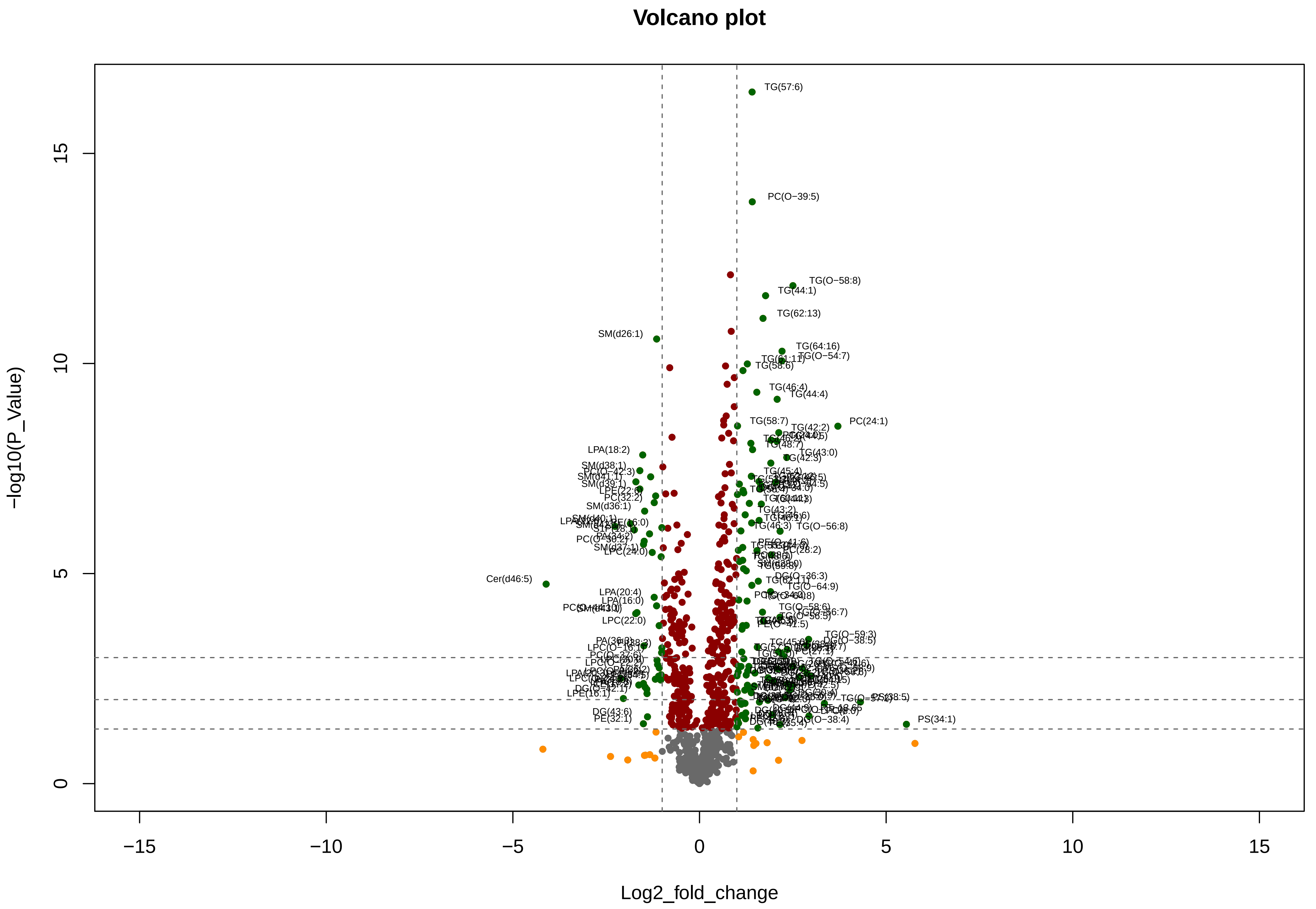

Volcano plot
−log10(P_Value)
Log2_fold_change
0
5
10
15
−15
−10
−5
0
5
10
15
TG(57:6)
PC(O−39:5)
TG(O−58:8)
TG(44:1)
TG(62:13)
SM(d26:1)
TG(64:16)
TG(O−54:7)
TG(58:6)
TG(46:4)
TG(44:4)
TG(58:7)
TG(42:2)
PC(24:1)
TG(48:7)
LPA(18:2)
TG(43:0)
TG(42:3)
SM(d38:1)
TG(45:4)
SM(d36:1)
TG(43:2)
TG(46:3)
TG(O−56:8)
SM(d37:1)
LPC(24:0)
Cer(d46:5)
TG(O−64:9)
LPA(20:4)
LPA(16:0)
LPC(22:0)
PE(O−41:5)
TG(O−59:3)
DG(O−38:5)
LPE(16:1)
DG(43:6)
PE(32:1)
PS(38:5)
PS(34:1)
DG(O−38:4)
